# Immunohistochemical Expression of Programmed Death-Ligand 1 (PD-L1) in Breast Carcinoma and Its Correlation With Estrogen Receptor, Progesterone Receptor and HER2/neu Status: A Single-Centre Study From Central India

**DOI:** 10.7759/cureus.114007

**Published:** 2026-08-05

**Authors:** Piyush Dubey, Parul Gupta, Manav Yadav

**Affiliations:** 1 Pathology, L.N. Medical College & J.K. Hospital, Bhopal, IND; 2 Community and Family Medicine, L.N. Medical College & J.K. Hospital, Bhopal, IND

**Keywords:** breast carcinoma, estrogen receptor, her2/neu, histological grade, immune checkpoint, immunohistochemistry, progesterone receptor, programmed death-ligand 1 (pd-l1)

## Abstract

Background: Programmed death-ligand 1 (PD-L1) has become both a therapeutic target and a predictive biomarker in breast cancer, yet reported expression rates vary widely and data from Indian populations remain limited. The primary aim of this study was to examine PD-L1 expression by immunohistochemistry in invasive breast carcinoma and to assess its relationship with histological grade and other clinicopathological variables. As a secondary aim, we evaluated the correlation of PD-L1 expression with estrogen receptor (ER), progesterone receptor (PR) and human epidermal growth factor receptor 2 (HER2/neu) status.

Materials and methods: This single-centre observational study analysed 61 histologically confirmed cases of invasive breast carcinoma of no special type at L.N. Medical College and J.K. Hospital, a tertiary care teaching hospital in Bhopal, central India. PD-L1 was scored using an immunohistochemical score (IHS) derived from staining intensity multiplied by the proportion of positive tumour cells, with an IHS of 3 or more taken as positive. ER and PR were regarded as positive at 1% or more nuclear staining, and HER2/neu was scored using American Society of Clinical Oncology/College of American Pathologists (ASCO/CAP) criteria; receptor status was assessable in 20 cases. Associations were tested using the chi-square and Fisher's exact tests, with a p-value below 0.05 considered significant.

Results: PD-L1 positivity was present in 14 of 61 tumours (23.0%). Expression rose steadily with histological grade, from 0% in grade I to 16.7% in grade II and 42.1% in grade III, and this association was statistically significant (χ² = 6.533, p = 0.038). PD-L1 positivity was numerically higher in ER-positive than in ER-negative tumours (44.4% vs. 9.1%), in PR-positive than in PR-negative tumours (44.4% vs. 9.1%) and in HER2/neu-positive than in HER2/neu-negative tumours (37.5% vs. 16.7%), but none of these differences reached significance (p = 0.127, 0.127 and 0.347, respectively). No significant association was found with age, tumour size, lymphovascular invasion, an in situ component, or pathological tumour (T) and node (N) stage.

Conclusions: PD-L1 was expressed in roughly one in four invasive breast carcinomas and was significantly associated with higher histological grade, supporting its link with poorly differentiated, biologically aggressive tumours. The trends observed with hormone receptor and HER2/neu status did not reach statistical significance and were limited by the small receptor-tested subset (n = 20), and these findings should therefore be regarded as hypothesis-generating. Larger, multicentre studies with complete molecular subtyping, standardised companion-diagnostic assays, and survival data are needed to confirm these relationships and to define the role of PD-L1 testing in selecting patients for immune checkpoint therapy.

## Introduction

Breast cancer is the most frequently diagnosed malignancy in women worldwide and remains a leading cause of cancer death [1]. In India, the disease now accounts for the largest share of cancers among women, and a substantial proportion of patients present at a younger age and with higher-grade, more advanced tumours than is typically seen in Western series [2,3]. Triple-negative disease, which carries a poorer prognosis and fewer targeted options, is also reported to be relatively common in Indian cohorts [4].

Breast cancer is biologically heterogeneous, and routine immunohistochemical assessment of estrogen receptor (ER), progesterone receptor (PR) and human epidermal growth factor receptor 2 (HER2/neu) underpins the working classification into hormone receptor-positive, HER2/neu-positive and triple-negative disease [5,6]. This classification continues to guide treatment: hormone receptor-positive tumours are managed with endocrine therapy and HER2/neu-positive tumours with HER2/neu-directed agents, whereas triple-negative tumours have, until recently, lacked an effective targeted approach [7].

The recognition that tumours co-opt immune checkpoints to evade the host immune response has changed this landscape. Programmed death-ligand 1 (PD-L1), expressed on tumour cells and on infiltrating immune cells, engages programmed cell death protein 1 (PD-1) on T lymphocytes and suppresses anti-tumour immunity [8]. Blocking the PD-1/PD-L1 axis restores T-cell activity, and immune checkpoint inhibitors are now incorporated into the management of PD-L1-positive triple-negative breast cancer, both in the metastatic and the early-disease settings [9,10].

Because the benefit of these agents is concentrated in PD-L1-positive tumours, reliable assessment of PD-L1 has acquired real clinical importance [11]. Reported positivity in breast cancer nonetheless spans a wide range, from roughly 10% to 60%, driven largely by differences in antibody clones, scoring algorithms and cut-offs, and compounded by variation in tumour subtype and patient ethnicity [12]. Its association with conventional prognostic factors, and with ER, PR and HER2/neu status, has likewise been inconsistent between studies.

Data on PD-L1 expression from Indian patients remain comparatively sparse. The primary aim of this study was to evaluate PD-L1 expression by immunohistochemistry in a series of invasive breast carcinomas at a tertiary care centre in central India and to examine its correlation with histological grade and other clinicopathological parameters. As a secondary aim, we assessed the association of PD-L1 expression with ER, PR and HER2/neu status.

## Materials and methods

Study design and setting

This was a single-centre, hospital-based observational study with both prospective and retrospective components, carried out in the Department of Pathology, L.N. Medical College and J.K. Hospital, a tertiary care teaching hospital in Bhopal, Madhya Pradesh, central India. The study was approved by the Institutional Ethics Committee of L.N. Medical College and Research Centre, Bhopal (approval number LNMC&RC/Dean/2024/Ethics/310, dated 10 May 2024) and was conducted in accordance with the Declaration of Helsinki. Written informed consent was obtained from all prospectively enrolled participants. The study was conducted over a period of 18 months.

Participants and specimens

A total of 61 histologically confirmed cases of breast carcinoma were included. The material comprised modified radical mastectomy specimens and tru-cut biopsies received during the study period. All histologically diagnosed breast carcinomas with available, adequate tissue were eligible. Cases with prior chemotherapy or radiotherapy, a concurrent second malignancy, or inadequate or non-representative tissue for immunohistochemistry were excluded.

Sample size

The minimum sample size was estimated from the proportion of PD-L1 positivity in grade III breast tumours reported by Muenst et al. [13], using the formula for the comparison of two proportions based on the risk ratio:

n = [Z(1−α/2) + Z(1−β)]² × [(1−p₁)/p₁ + (1−p₂)/p₂] ÷ [ln(p₁/p₂)]²,

where Z(1−α/2) = 1.96 (two-sided α = 0.05), Z(1−β) = 1.28 (β = 0.10; power = 90%), p₁ = 0.314 (proportion of PD-L1-positive grade III tumours) and p₂ = 0.686 (proportion of PD-L1-negative tumours). Substituting these values, with a 10% allowance for anticipated data loss, yielded a minimum required sample size of 51; 61 cases were ultimately enrolled.

Histopathology and grading

All specimens underwent routine processing, and 4-5 µm haematoxylin and eosin-stained sections were examined. Tumours were classified according to the World Health Organization (WHO) classification of breast tumours [14]; all 61 cases were invasive breast carcinoma of no special type. Histological grade was assigned using the Nottingham (Elston-Ellis modified Scarff-Bloom-Richardson) system [15], scoring tubule formation, nuclear pleomorphism and mitotic rate, with the summed score defining grade I (3-5), grade II (6-7) or grade III (8-9). In resection specimens, pathological tumour (pT) and nodal (pN) stage were assigned according to the American Joint Committee on Cancer (AJCC) staging criteria [16].

Immunohistochemistry

Five-micrometre sections from formalin-fixed, paraffin-embedded blocks were mounted on coated slides. After dewaxing and rehydration, antigen retrieval was carried out by microwaving in citrate buffer (pH 6.0), and endogenous peroxidase was quenched with hydrogen peroxide in methanol. Sections were then incubated with a ready-to-use rabbit monoclonal anti-PD-L1 antibody (clone IHC411, catalogue number AN921; BioGenex, Fremont, CA, USA). Bound antibody was detected with a horseradish peroxidase-conjugated polymer detection system using 3,3′-diaminobenzidine as the chromogen (Polymer-HRP/DAB, BioGenex, Fremont, CA, USA), and the sections were counterstained with haematoxylin. Immunohistochemistry for ER, PR and HER2/neu was performed on separate sections by the same technique. A positive control was included in every run, and a negative control was prepared by omitting the primary antibody.

Scoring

PD-L1 was assessed using an immunohistochemical score (IHS) calculated as the product of staining intensity (0 = none, 1 = light yellow, 2 = brownish-yellow, 3 = dark brown) and the proportion of positive tumour cells (1 = 10% or less, 2 = 10-50%, 3 = more than 50%) [17]. Cytoplasmic and membranous staining of tumour cells was evaluated across five randomly selected high-power fields, and an IHS of 3 or more was classified as positive. ER and PR were interpreted according to ASCO/CAP recommendations, with 1% or more nuclear positivity regarded as positive [18]. HER2/neu was scored by the ASCO/CAP membrane-staining criteria, with scores of 0 and 1+ taken as negative and 3+ as positive [19]. Receptor status was assessable in 20 of the 61 cases.

Statistical analysis

Data were analysed using R version 4.5.3 (R Foundation for Statistical Computing, Vienna, Austria). Continuous variables are expressed as mean ± standard deviation and categorical variables as frequencies and percentages. Associations between PD-L1 expression and categorical clinicopathological variables were tested using the Pearson chi-square test; the Fisher exact test was applied where expected cell counts were small, specifically to pN status and to the ER, PR and HER2/neu comparisons. Because histological grade is an ordered variable, the Cochran-Armitage test for linear trend was used as a supporting analysis of the grade-PD-L1 relationship. The independent-samples t-test was used to compare continuous variables between PD-L1-positive and PD-L1-negative groups. Test statistics (chi-square or t values, with their degrees of freedom) are reported alongside the corresponding p-values. A two-tailed p-value below 0.05 was considered statistically significant.

## Results

The 61 patients ranged in age from 20 to 81 years, with a mean of 52.61 ± 12.75 years; the majority (44/61, 72.1%) were older than 45 years. Right-sided tumours predominated (40/61, 65.6%), and most specimens were mastectomies (43/61, 70.5%). All tumours were invasive carcinoma of no special type. Grade II tumours were the most frequent (36/61, 59.0%), followed by grade III (19/61, 31.2%) and grade I (6/61, 9.8%), such that nine of every 10 tumours were of intermediate or high grade. A ductal carcinoma in situ (DCIS) component was present in 28 cases (45.9%). Among the 43 resection specimens, lymphovascular invasion was identified in 27 (62.8%), the mean tumour size was 3.95 ± 2.17 cm, pT2 was the most common stage (20/43, 46.5%), and 22 of 43 (51.2%) were node-positive. Receptor testing, available in 20 cases, showed ER positivity in nine (45.0%), PR positivity in nine (45.0%) and HER2/neu positivity in eight (40.0%). These features are summarised in Table 1.

**Table 1:** Demographic and clinicopathological characteristics of the study population Percentages are calculated on the whole cohort of 61 cases unless a different denominator is given in parentheses. The denominators differ because pathological tumour (pT) stage, pathological nodal (pN) status and lymphovascular status could be assessed only in the 43 resection specimens, and receptor status only in the 20 cases with sufficient residual tissue for further immunohistochemistry. DCIS, ductal carcinoma in situ; ER, estrogen receptor; HER2/neu, human epidermal growth factor receptor 2; PR, progesterone receptor

Variable	Frequency (n)	Percentage (%)
Age ≤45 years	17	27.9
Age >45 years	44	72.1
Laterality: right	40	65.6
Laterality: left	21	34.4
Specimen: modified radical mastectomy	43	70.5
Specimen: tru-cut biopsy	18	29.5
Grade I (well differentiated)	6	9.8
Grade II (moderately differentiated)	36	59.0
Grade III (poorly differentiated)	19	31.2
DCIS component present	28	45.9
Lymphovascular invasion present (n = 43)	27	62.8
pT1–pT2 (n = 43)	31	72.1
pT3–pT4 (n = 43)	12	27.9
Node-positive, pN1–pN3 (n = 43)	22	51.2
ER positive (n = 20)	9	45.0
PR positive (n = 20)	9	45.0
HER2/neu positive (n = 20)	8	40.0

PD-L1 expression

PD-L1 was expressed in 14 of the 61 tumours, an overall positivity rate of 23.0%; the remaining 47 (77.0%) were negative. Positive staining appeared as cytoplasmic to membranous yellow-to-brown reactivity in tumour cells.

PD-L1 and clinicopathological parameters

Of all the variables examined, only histological grade was significantly associated with PD-L1 expression (χ² = 6.533, df = 2, p = 0.038). Positivity increased stepwise with grade, from none of the six grade I tumours, to 16.7% (6/36) of grade II and 42.1% (8/19) of grade III tumours; the test for linear trend across the three grades was also significant (Cochran-Armitage χ² = 6.402, df = 1, p = 0.011), and in pairwise testing the difference was driven by the contrast between grade II and grade III (χ² = 4.241, df = 1, p = 0.039). PD-L1-positive cases also tended to be older (mean age 57.36 ± 14.83 versus 51.19 ± 11.87 years) and showed a declining frequency with advancing pT stage (36.4% at pT1 falling to 0% at pT4), but neither trend reached significance. No association was found with laterality, tumour size, lymphovascular invasion, the DCIS component, or pN status. These results are presented in Table 2.

**Table 2:** Association of PD-L1 expression with clinicopathological parameters *Statistically significant (p < 0.05). Format: for the categorical variables, values are n (%), where n is the number of cases and the percentage is the proportion of that category which was PD-L1-positive or PD-L1-negative; the two percentages in each row therefore sum to 100%, and the "Assessable cases (n)" column gives the denominator. For the two continuous variables (marked a), values are mean ± standard deviation and the "Assessable cases (n)" column gives the total number of cases in which the variable could be measured. Why the denominators differ: age, laterality, histological grade and the DCIS component were assessable in all 61 cases (14 PD-L1-positive, 47 PD-L1-negative), whereas tumour size, lymphovascular invasion, pT stage and pN status could be determined only in the 43 resection specimens (10 PD-L1-positive, 33 PD-L1-negative); tru-cut biopsies do not permit assessment of these variables. a Continuous variable, expressed as mean ± standard deviation and compared using the independent-samples t-test. b The p-value for nodal status was obtained with the Fisher exact test, because expected cell counts were below five; the Pearson chi-square statistic is shown alongside for reference. All other categorical variables were compared using the Pearson chi-square test. As a supporting analysis, the Cochran-Armitage test for linear trend across the three histological grades was also significant (χ² = 6.402, df = 1, p = 0.011). DCIS, ductal carcinoma in situ; PD-L1, programmed death-ligand 1; pN, pathological nodal stage; pT, pathological tumour stage

Parameter	Category	Assessable cases (n)	PD-L1 positive	PD-L1 negative	Test statistic	p-value
Age, years ^a^	—	61	57.36 ± 14.83	51.19 ± 11.87	t = 1.611 (df = 59)	0.113
Laterality	Right	40	10 (25.0)	30 (75.0)	χ² = 0.276 (df = 1)	0.599
	Left	21	4 (19.0)	17 (81.0)		
Histological grade	Grade I	6	0 (0.0)	6 (100.0)	χ² = 6.533 (df = 2)	0.038*
	Grade II	36	6 (16.7)	30 (83.3)		
	Grade III	19	8 (42.1)	11 (57.9)		
DCIS component	Present	28	6 (21.4)	22 (78.6)	χ² = 0.068 (df = 1)	0.795
	Absent	33	8 (24.2)	25 (75.8)		
Tumour size, cm ^a^	—	43	3.07 ± 1.83	4.22 ± 2.21	t = 1.494 (df = 41)	0.143
Lymphovascular invasion	Present	27	6 (22.2)	21 (77.8)	χ² = 0.043 (df = 1)	0.835
	Absent	16	4 (25.0)	12 (75.0)		
pT stage	pT1–pT2	31	9 (29.0)	22 (71.0)	χ² = 2.077 (df = 1)	0.150
	pT3–pT4	12	1 (8.3)	11 (91.7)		
pN status	Positive	22	4 (18.2)	18 (81.8)	χ² = 0.650 (df = 1) ^b^	0.488 ^b^
	Negative	21	6 (28.6)	15 (71.4)		

PD-L1 and ER, PR and HER2/neu status

In the 20 cases with receptor data, PD-L1 positivity was more frequent in hormone receptor-positive and HER2/neu-positive tumours. Four of the nine ER-positive tumours (44.4%) were PD-L1-positive, compared with one of the 11 ER-negative tumours (9.1%); the figures for PR were identical, reflecting the usual co-expression of ER and PR. PD-L1 positivity was seen in three of the eight HER2/neu-positive tumours (37.5%) versus two of the 12 HER2/neu-negative tumours (16.7%). Viewed the other way round, four of the five PD-L1-positive cases (80.0%) were ER-positive, compared with five of the 15 PD-L1-negative cases (33.3%). Despite these consistent trends, none of the associations reached statistical significance (ER and PR, p = 0.127; HER2/neu, p = 0.347), as detailed in Table 3.

**Table 3:** Association of PD-L1 expression with ER, PR and HER2/neu status Format: values are n (%), where n is the number of cases in that receptor category and the percentage is the proportion of that category which was PD-L1-positive or PD-L1-negative; the two percentages in each row sum to 100%. Why the denominators differ: receptor status could be assessed only in the 20 of 61 cases with sufficient residual tissue (five PD-L1-positive and 15 PD-L1-negative). The same 20 cases contribute to every receptor, so the two "Assessable cases (n)" values in each pair of rows sum to 20; they differ between rows only because the number of receptor-positive and receptor-negative tumours differs for each marker. All p-values were obtained with the Fisher exact test, which was used because expected cell counts were below five; the corresponding Pearson chi-square statistic is shown alongside for reference. None of the associations was statistically significant. ER, estrogen receptor; HER2/neu, human epidermal growth factor receptor 2; PD-L1, programmed death-ligand 1; PR, progesterone receptor

Receptor	Status	Assessable cases (n)	PD-L1 positive	PD-L1 negative	Test statistic	p-value
ER	Positive	9	4 (44.4)	5 (55.6)	χ² = 3.300 (df = 1)	0.127
	Negative	11	1 (9.1)	10 (90.9)		
PR	Positive	9	4 (44.4)	5 (55.6)	χ² = 3.300 (df = 1)	0.127
	Negative	11	1 (9.1)	10 (90.9)		
HER2/neu	Positive	8	3 (37.5)	5 (62.5)	χ² = 1.111 (df = 1)	0.347
	Negative	12	2 (16.7)	10 (83.3)		

## Discussion

In this series of 61 invasive breast carcinomas, PD-L1 was expressed in just under a quarter of tumours and was significantly more frequent in higher-grade disease. The numerically higher positivity seen in ER-positive, PR-positive and HER2/neu-positive tumours did not reach statistical significance, and no relationship was found with tumour size, stage or lymphovascular invasion.

Our positivity rate of 23.0% sits comfortably within the broad 10-60% range reported in the literature. Much of this spread reflects methodological rather than biological differences: the antibody clone and platform, the cut-off and the cell compartment scored, and the mix of tumour subtypes in a given cohort [12]. Schalper et al. found PD-L1 in around 20-30% of breast carcinomas and linked it closely to tumour-infiltrating lymphocytes [20], while subtype-enriched series report considerably higher figures, with Sabatier et al. describing enrichment in basal-like tumours [21] and Parvathareddy et al. reporting positivity of roughly 40-50% in triple-negative disease [22]. The intermediate rate in our mixed, predominantly hormone receptor-containing cohort is therefore expected.

The clearest signal in our data was the association between PD-L1 and histological grade, with positivity rising from none of the grade I tumours to 42.1% of grade III tumours. Several other studies have reported the same pattern. Ghebeh et al. were among the first to link PD-L1 with high-risk pathological features including grade [23], and a later meta-analysis by Wang et al. confirmed a significant relationship between PD-L1 expression and high-grade disease [24]. A plausible explanation is adaptive immune resistance: high-grade, genomically unstable tumours tend to provoke a brisker immune response, and the resulting interferon-γ signalling upregulates PD-L1 as a counter-regulatory mechanism [25]. On this view PD-L1 is less a passive marker of aggression than a readout of an active, ongoing tumour-immune interaction.

The absence of any association with tumour size, pT stage or pN status echoes the findings of Baptista et al., who likewise found no consistent correlation between PD-L1 and conventional tumour-node-metastasis (TNM) parameters [26]. These findings suggest that PD-L1 expression tracks the intrinsic biology of the tumour rather than the anatomical extent of disease.

The relationship with hormone receptor status is where our results depart from much of the published work. We observed a trend toward higher PD-L1 expression in ER-positive and PR-positive tumours, whereas most large series report the opposite, with PD-L1 enriched in ER-negative, triple-negative and HER2/neu-enriched tumours [21,27]. This discordance must be interpreted with considerable caution. Receptor data were available in only 20 cases, rendering the comparison substantially underpowered and highly susceptible to chance variation; with four of the five PD-L1-positive cases happening to be hormone receptor-positive, a single reclassified case would materially alter the proportions. It is also true that a subset of immune-rich luminal tumours can express PD-L1, as Park et al. have noted [28], so the finding is not biologically implausible, though it should be regarded as strictly hypothesis-generating and requires confirmation in a larger, fully subtyped cohort before any clinical or biological inferences are drawn.

PD-L1 positivity was also higher in HER2/neu-positive tumours, again without reaching significance. While this observation is consistent with the recognised crosstalk between HER2/neu signalling and the PD-1/PD-L1 pathway [29], and with reports that PD-L1 expression in HER2/neu-positive disease is associated with immune infiltration [30], the small number of HER2/neu-positive cases (n = 8) in our receptor subset precludes any firm conclusion, and this trend should be interpreted as preliminary.

From a practical standpoint, the benefit of immune checkpoint inhibitors in breast cancer is largely confined to PD-L1-positive tumours, as shown for atezolizumab in metastatic triple-negative disease and for pembrolizumab in the early-disease setting [9,10], and meta-analytic data confirm that response to PD-1/PD-L1 blockade is significantly greater in PD-L1-positive cancers [11]. While a positivity rate of 23% suggests that a subset of patients in this population might, in principle, be considered for such therapy, particularly within the triple-negative and HER2/neu-positive subgroups, this possibility remains speculative in the absence of molecular subtyping and outcome data. Realising that potential would also depend on harmonised testing, since assay and scoring differences materially affect which tumours are called positive [12].

The strengths of this study include a real-world cohort drawn from routine practice, uniform processing and a single, consistently applied scoring scheme. Its limitations, however, are substantial and warrant explicit discussion. First, the overall sample size was modest (n = 61), and the receptor-tested subset was small (n = 20), which considerably limited statistical power for the ER, PR and HER2/neu comparisons and precluded full molecular subtyping into luminal A, luminal B, HER2/neu-enriched and triple-negative categories; this is the principal reason that the observed trends with receptor status must be considered hypothesis-generating rather than definitive. Second, the antibody clone used (IHC411, BioGenex) is not among those validated in the registrational checkpoint-inhibitor trials (SP142 for atezolizumab, 22C3 for pembrolizumab, SP263), and PD-L1 positivity was defined by an immunohistochemical score rather than by the tumour proportion score or the combined positive score employed in those trials; our positivity rate is therefore not directly interchangeable with that of the companion-diagnostic assays, and the clinical applicability of our findings to checkpoint-inhibitor selection remains uncertain. Third, the study was confined to a single centre, tumour-infiltrating lymphocytes were not formally quantified, and no survival or follow-up data were available, so the prognostic and predictive implications of PD-L1 in this cohort could not be assessed. Larger, multicentre studies with standardised assays, complete molecular subtyping and outcome data will be needed to settle the relationship between PD-L1 and hormone receptor and HER2/neu status, and to establish its prognostic and predictive value in Indian patients.

## Conclusions

PD-L1 was expressed in approximately one in four invasive breast carcinomas in this central Indian cohort and was significantly associated with higher histological grade, supporting its role as a marker of poorly differentiated, biologically aggressive tumours. Although PD-L1 positivity was numerically higher in ER-positive, PR-positive and HER2/neu-positive tumours, these associations were not statistically significant and were substantially constrained by the small receptor-tested subset; they should therefore be regarded as preliminary observations requiring confirmation. Given the established predictive role of PD-L1 in immune checkpoint therapy, and the relatively high frequency of high-grade and triple-negative disease in Indian women, systematic assessment of PD-L1 may potentially help identify patients who could benefit from immunotherapy, although this remains to be validated in prospective studies with standardised companion-diagnostic assays. Larger multicentre studies incorporating standardised scoring, complete molecular subtyping and survival analysis are warranted to confirm these findings.
